# Increased Jab1/COPS5 is associated with therapeutic response and adverse outcome in lung cancer and breast cancer patients

**DOI:** 10.18632/oncotarget.22146

**Published:** 2017-10-27

**Authors:** Junna Hou, Guohong Liu, Yufen Yuan, Dong Wang, Pengfei Jiao, Lihua Xing, Yunbao Pan

**Affiliations:** ^1^ Department of Pulmonary Medicine, The First Affiliated Hospital of Zhengzhou University, Zhengzhou, Henan 450000, China; ^2^ School of Materials Science and Engineering and School of Electronics and Information Technology, Sun Yat-Sen University, Guangzhou, Guangdong 510275, China; ^3^ Department of Pathology, Anyang Tumor Hospital, Anyang, Henan 455000, China; ^4^ Department of Laboratory Medicine, Zhongnan Hospital of Wuhan University, Wuhan University, Wuhan, Hubei 430071, China; ^5^ Breast Tumor Center, Sun Yat-Sen Memorial Hospital, Sun Yat-Sen University, Guangzhou, Guangdong 510080, China

**Keywords:** breast cancer, lung cancer, Jab1, biomarkers, immunohistochemistry

## Abstract

Adjuvant chemotherapy has been established as standard treatment for advanced cancer among multidisciplinary therapies. A simple and instructive biomarker for therapeutic response and recurrence is needed to evaluate the therapeutic effect. Jab1/COPS5 level has been shown to be associated with tumor progression and poor outcomes in many types of cancer patients. This study aims to further evaluate the clinical and prognostic value of Jab1/COPS5 level as a biomarker in lung and breast cancer patients receiving adjuvant chemotherapy. In this study, data of 88 lung cancer and 76 breast cancer patients were retrospectively collected and analyzed to identify the relationship between the Jab1/COPS5 level and the clinical progression and outcome of these patients. Lung cancer patients with increased Jab1/COPS5 level tend to be non-responsive to chemotherapy. Relapsed breast cancer patients had an increased Jab1/COPS5 level and breast cancer patients with increased Jab1/COPS5 level had significantly shorter disease-free survival and overall survival. In a multivariate survival analysis, histological type and Jab1/COPS5 were associated with disease-free survival and overall survival. The Jab1/COPS5 level was found to be a possible biomarker for clinical response to chemotherapy in lung cancer patients and for postoperative relapse in breast cancer patients who received adjuvant chemotherapy. In conclusion, this study identified Jab1/COPS5 as novel prognostic markers for lung cancer and breast cancer.

## INTRODUCTION

Precision medicine needs to select patients with the right target to achieve appropriate treatment approach and prognosis [1]. In the past decades, several biomarkers were discovered, for example, epidermal growth factor receptor (EGFR) in lung cancer patients [2] and hormone receptor (HR), human epidermal growth factor receptor 2 (HER2), estrogen (ER), progesterone receptor (PR) in breast cancer patients [3, 4]. Another potential target in cancer is Jab1. Jab1 was initially identified as c-Jun activation domain-binding protein-1 (Jab1) [5] and subsequently discovered to be the fifth component of the constitutive photomorphogenic-9 signalosome (COPS5). This protein regulates a variety of cellular and developmental processes, including signal transduction, cells proliferation, cell cycle, apoptosis, DNA damage response (DDR) and tumorigenesis [6]. Increasing evidence indicates that dysregulation of Jab1/COPS5 activity contributes to tumorigenesis, which is functionally associated with cancer-related genes including p27 [7], p57 [8], SMAD4 [9], NcoR [10], Trx [11] and so on [12]. Studies on Jab1/COPS5 has focused on identification of tumor types that show Jab1/COPS5 overexpression, including colon, ovarian, lung and breast cancer [6].

Lung cancer is the most common cancer worldwide with a poor five-year survival estimated at 15%. This disease can be divided in NSCLC, contributing to 85% of all lung cancers, and small cell lung cancer (SCLC) [13]. Tumor-targeted therapies have already been extensively studied in lung cancer patients, which resulted in implementation of such therapies, for example, erlotinib, bevacizumab and gefitinib, in the treatment guideline of lung cancer [14]. Although Jab1/COPS5 was found to be overexpressed in lung cancer, its role on therapeutic response remains to be determined.

In breast cancer, treatment is already personalized by detecting molecular markers [4]. The outcome of patients is significantly improved by targeted therapy focused on HR positive, or HER2 positive cancers. However, triple negative breast cancers (TNBC) account for 15% of all breast cancers, defect in either ER/PR or HER2. TNBC has the worst prognosis amongst all breast cancers [15]. Previous studies reported Jab1/COPS5 overexpressed in breast cancers and therefore Jab1/COPS5 could be an interesting target for breast cancer patients [16]. However, the role of Jab1/COPS5 on breast cancer relapse needs to be elucidated.

The aim of the current study is to examine Jab1/COPS5 expression on both lung and breast cancers and to investigate its role on therapeutic response and cancer relapse.

## RESULTS

### Patient characteristics and demographics

**Figure 1:**
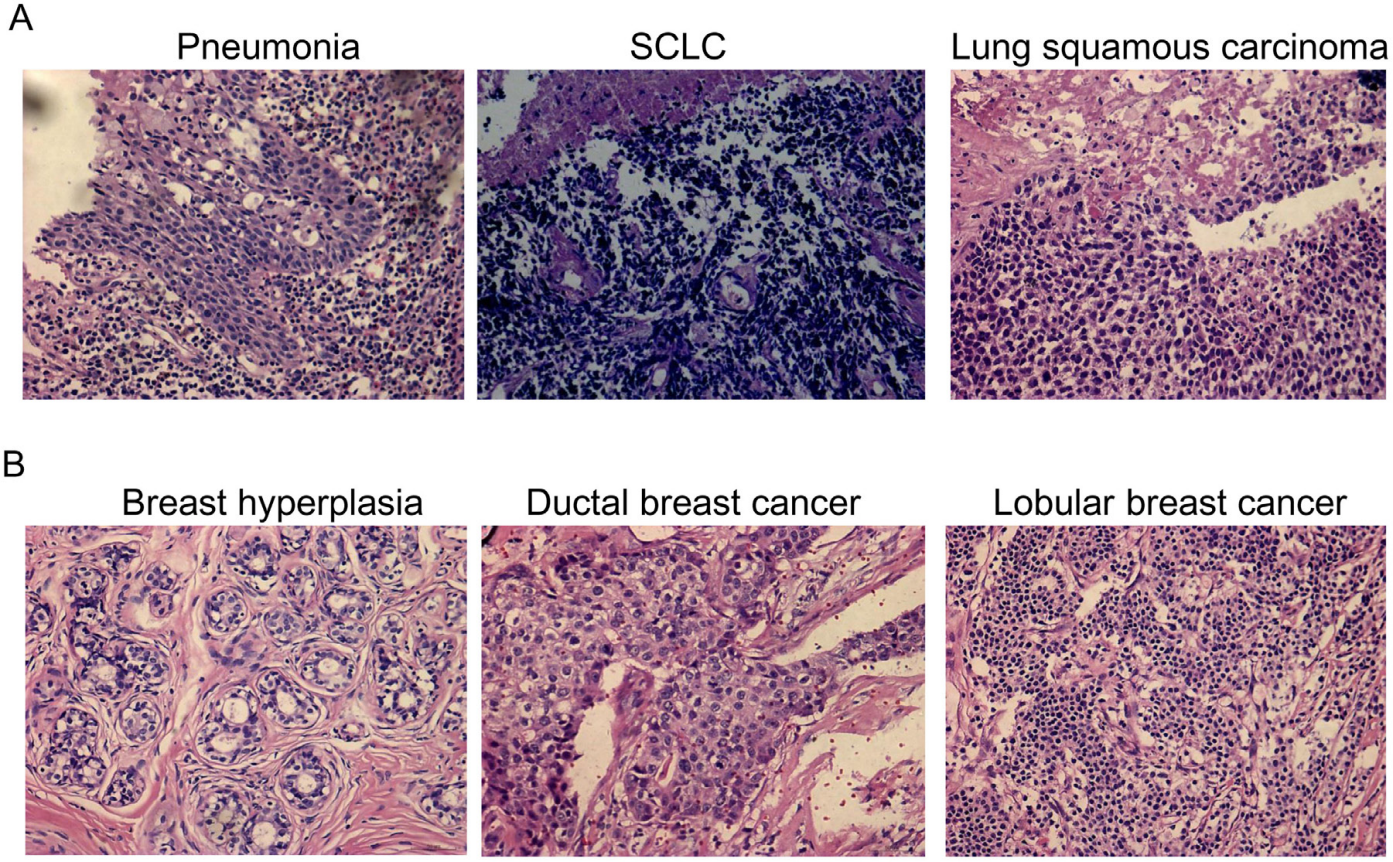
HE staining of cancer tissue and non-cancerous tissue **(A)** Pneumonia, small cell lung cancer (SCLC) and lung squamous carcinoma. **(B)** Breast hyperplasia, ductal breast cancer and lobular breast cancer.

To analyze the Jab1/COPS5 expression patterns, we used 88 lung cancer tissue samples (mean age, 59 years; range, 33–81 years) and 76 breast cancer tissue samples (mean age, 49 years; range, 25–80 years). Characteristics of noncancerous and cancerous patients were summarized in Supplementary Table 1. HE staining of the patients’ tissue is showed in Figure 1.

### Association between Jab1/COPS5 and clinicopathological parameters

**Figure 2:**
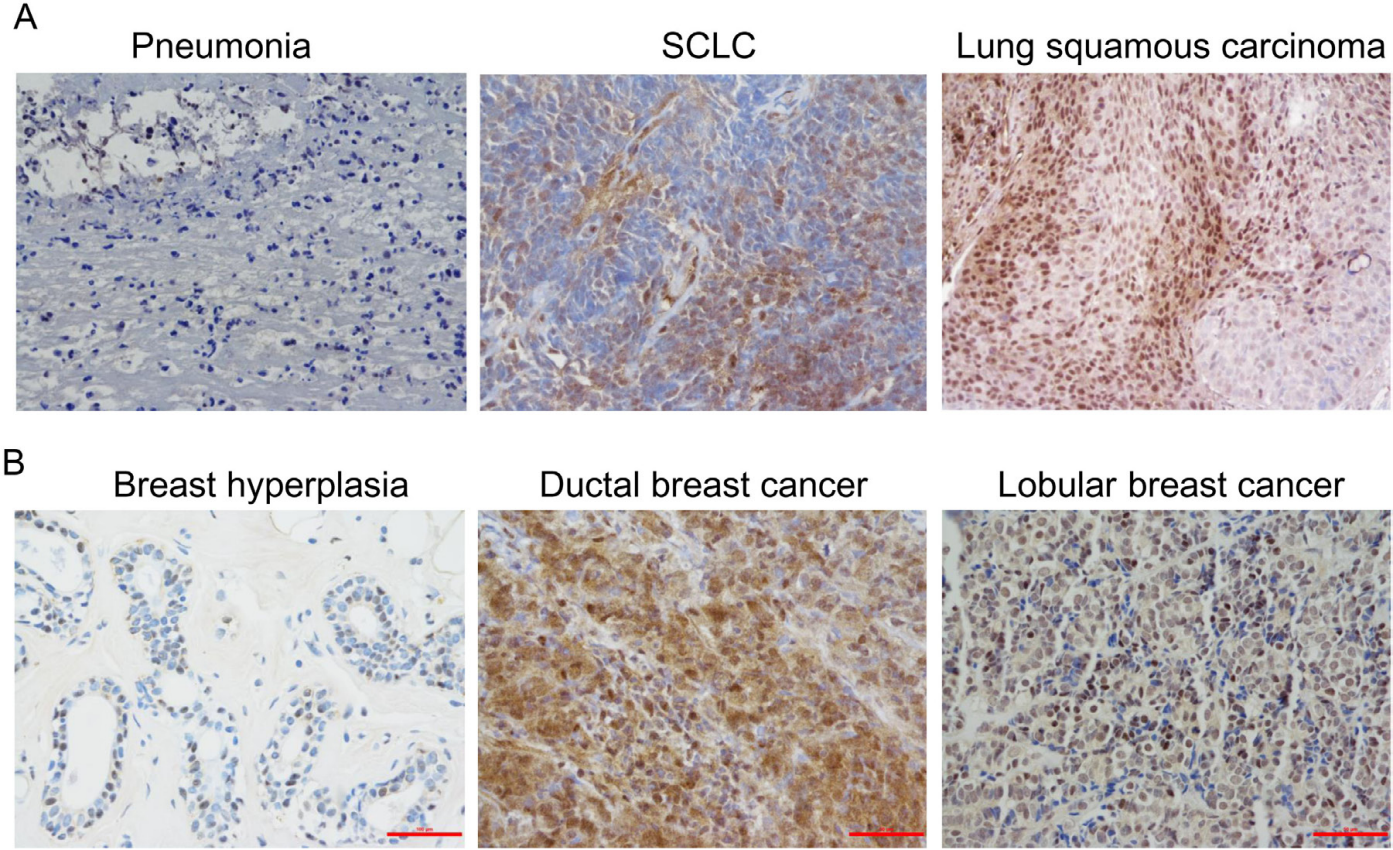
Immunohistochemical analysis of Jab1/COPS5 in cancer tissues **(A)** Pneumonia (left), small cell lung cancer (SCLC) (middle) and lung squamous carcinoma (right). **(B)** Breast hyperplasia (left), ductal breast cancer (middle) and lobular breast cancer (right).

**Figure 3:**
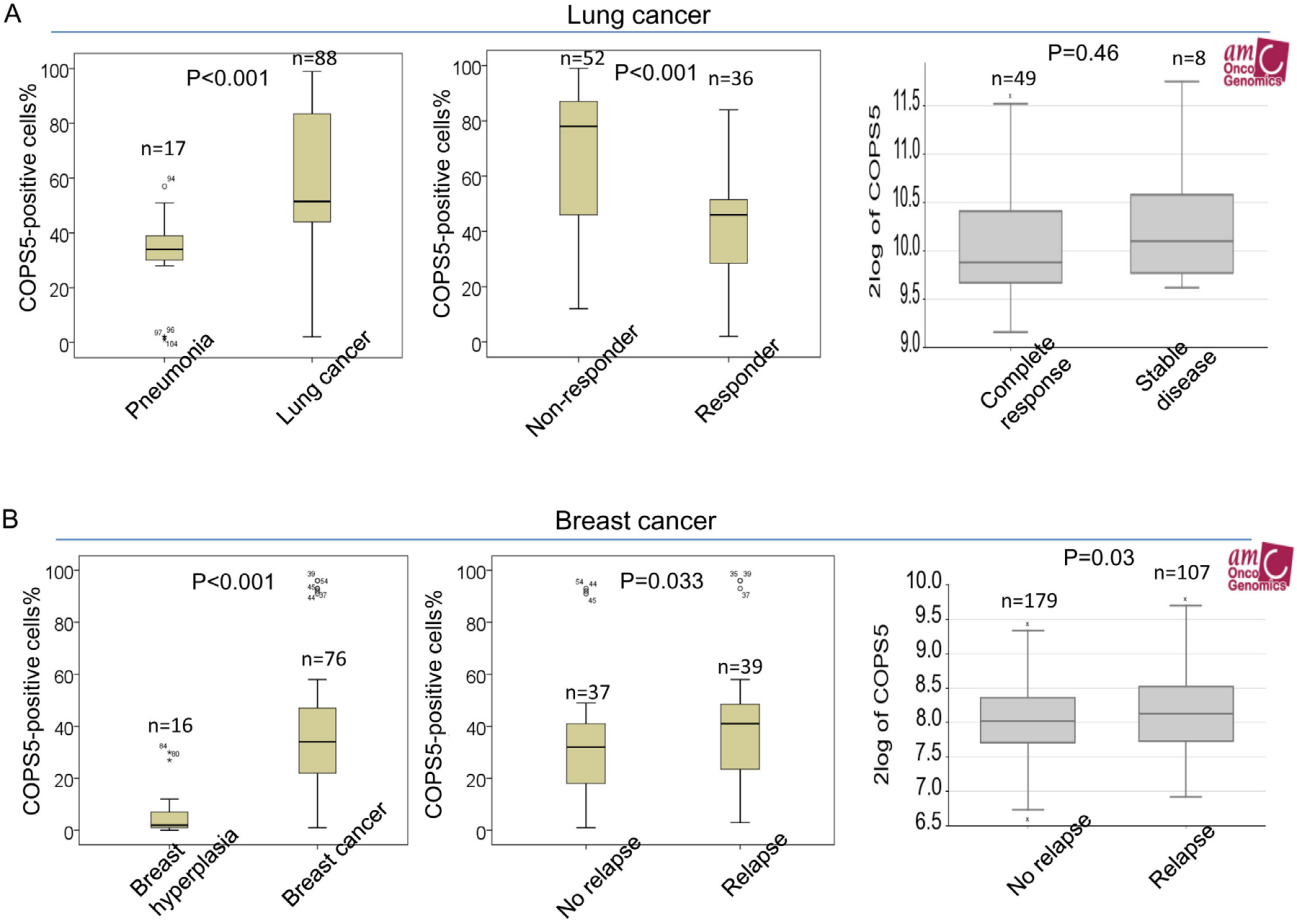
Jab1/COPS5 is overexpressed in cancer tissues **(A)** The percentages of positive Jab1/COPS5 cell in pneumonia and lung cancer (left), in lung cancer patients with non-responsive and responsive to chemotherapy (middle). (Right) Jab1/COPS5 gene expression in lung cancer patients with complete response or stable disease was plotted using the Oncomine gene expression tool (https://www.oncomine.com). The clinical data were downloaded from Oncomine Data Portal. **(B)** The percentages of positive Jab1/COPS5 cell in breast hyperplasia and breast cancer (left), in breast cancer patients without relapse or with relapse (middle). (Right) Jab1/COPS5 gene expression in breast cancer patients without relapse or with relapse was plotted using the Oncomine gene expression tool.

The expression of Jab1/COPS5 in lung cancer or breast cancer was detected by IHC (Figure 2). It was demonstrated that COPS5-positive cells in cancer tissue were significantly higher than those in non-cancerous tissue (Figure 2 and Figure 3). Correlations between COPS5 positive cases and clinicopathological parameters were summarized in Table 1 and Table 2. COPS5 expression was correlated with histological type (p=0.035), tumor stage (p=0.017), lymph node metastasis (p=0.016), distant metastasis (p=0.007) and hydrothorax status (p=0.039) in lung cancer patients (Table 1). However, COPS5 expression was correlated with ER status (p<0.001) and PR status (p=0.013) in breast cancer patients (Table 2).

**Table 1:** Demographic and characteristics of Jab1 in lung cancer patients (n=88)

Variable	Total n=88	Low COPS5	High COPS5	p
Age				
<60	39	13(33.3%)	26(66.7%)	0.249
≥60	49	12(24.5%)	37(75.5%)	
Sex				
Male	67	19(28.4%)	48(71.6%)	0.594
Female	21	6(28.6%)	15(71.4%)	
Histological type				
SCLC	31	13(41.9%)	18(58.1%)	0.035
NSCLC	57	12(21.1%)	45(78.9%)	
Smoking				
No	37	11(29.7%)	26(70.3%)	0.500
Yes	51	14(27.5%)	37(72.5%)	
Stage				
I	9	1(11.1%)	8(88.9%)	0.017
II	9	1(11.1%)	8(88.9%)	
III	11	0(0%)	11(100%)	
IV	59	23(39.0%)	36(61.0%)	
Tumor size				
T1	29	6(20.7%)	23(79.3%)	0.186
T2	44	13(29.5%)	31(70.5%)	
T3	12	6(50.0%)	6(50.0%)	
T4	3	0(0%)	3(100%)	
LN metastasis				
No	22	2(9.1%)	20(90.9)	0.016
Yes	66	23(34.8%)	43(65.2%)	
Distant metastasis				
No	37	5(13.5%)	32(86.5%)	0.007
Yes	51	20(39.2%)	31(60.8%)	
Ki-67				
<14%	2	1(50.0%)	1(50.0%)	0.490
≥14%	86	24(27.9%)	62(72.1%)	
Hydrothorax				
No	63	14(22.2%)	49(77.8%	0.039
Yes	25	11(40.0%)	14(56.0%)	

**Table 2:** Demographic and characteristics of Jab1 in breast cancer patients (n=76)

Variable	Total n=76	Low Jab1	High Jab1	p
Age				
<60	64	24(37.5%)	40(62.5%)	0.785
≥60	12	5(41.7%)	7(58.3%)	
Histological type				
Ductal	70	28(40.0%)	42(60.0%)	0.398
Lobular	6	1(16.7%)	5(83.3%)	
Stage				
I	9	4(44.4%)	5(55.6%)	0.211
II	46	14(30.4%)	32(69.6%)	
III	21	11(52.4%)	10(47.6%)	
Grade				
G1	6	2(33.3%)	4(66.7%)	0.758
G2	50	18(36.0)	32(64.0%)	
G3	20	9(45.0%)	11(55.0%)	
Tumor size				
T1	8	4(50%)	4(50%)	0.087
T2	46	13(28.3%)	33(71.7%)	
T3	22	12(54.5%)	10(45.5%)	
LN metastasis				
Yes	63	25(39.7%)	38(60.3)	0.547
No	13	4(30.8%)	9(69.2%)	
Distant metastasis				
No	75	28(37.3%)	47(62.7%)	0.382
Yes	1	1(100.0%)	0 (0.0%)	
Ki-67				
<14%	37	15(40.5%)	22(59.5%)	0.677
≥14%	39	14(35.9%)	25(64.1%)	
HER2				
negative	36	12(33.3%)	24(66.7%)	0.411
positive	40	17(42.5%)	23(57.5%)	
ER				
negative	38	22(57.9%)	16(42.1%)	<0.001
positive	38	7(18.4%)	31(81.6%)	
PR				
negative	44	22(50.0%)	22(50.0%)	0.013
positive	32	7(21.9%)	25(78.1%)	
Family history				
Yes	22	12(54.5%)	10(45.5%)	0.060
No	54	17(31.5%)	37(68.5%)	
Menopause				
Yes	32	15(46.9%)	17(53.1%)	0.182
No	44	14(31.8%)	30(68.2%)	

### Jab1/COPS5 predicts treatment response in lung cancer and relapse in breast cancer patients

It was found that lung cancer patients with no chemotherapy response had significant higher COPS5 expression than those with response (Figure 3A, middle). In agreement with our immunohistochemistry findings, the data from online database R2 indicated that COPS5 mRNA expression levels in lung cancer patients with stable disease are higher than those with complete response (Figure 3A, right). However, we did not obtain statistical difference from the online data which may be attributed to a small sample size. Logistic regression was further used to identify risk factors impacting treatment response in lung cancer. Variables examined in multivariate analysis and significant variables were shown in Table 3. In the logistic regression, lymph node involvement, distant metastasis and COPS5 were associated with treatment response with a more prominent predictive effect (P < 0.05) (Table 3).

**Table 3:** Logistic regression analysis of factors predicting treatment response in lung cancer patients

Parameters	B	Wald	Sig.	Exp (B)	95% CI
Age (≥60y)	0.523	0.569	0.451	1.689	0.433-6.572
Sex	0.977	0.819	0.365	2.655	0.320-22.006
Histological type	1.125	2.339	0.126	3.082	0.728-13.038
Stage	0.162	0.063	0.801	1.176	0.333-4.145
Tumor size	-0.039	0.006	0.940	0.962	0.346-2.675
LN metastasis (+)	-2.288	6.238	0.013	0.101	0.017-0.611
Distant metastasis (+)	-1.622	5.411	0.020	0.197	0.050-0.775
Smoking (+)	0.941	1.803	0.179	2.563	0.649-10.124
Hydrothorax (+)	-0.288	0.121	0.728	0.750	0.148-3.805
Ki-67 (≥ 14%)	0.020	1.406	0.236	1.021	0.987-1.056
Jab1/COPS5	-0.101	18.083	0.000	0.904	0.862-0.947

LN: lymph node.

It was also demonstrated that recurrent breast cancer patients had significant higher COPS5 expression than those primary tumors (Figure 3B, middle), which was in agreement with analysis from online database that COPS5 mRNA expression levels in recurrent breast cancer patients are significant higher than non-recurrent tumors (Figrue 3B, right). Logistic regression was also used to identify risk factors impacting recurrence in breast cancer. In the logistic regression, Ki-67 and COPS5 were associated with relapse (P < 0.05) (Table 4).

**Table 4:** Logistic regression analysis of factors predicting recurrence in breast cancer patients

Parameters	B	Wald	Sig.	Exp (B)	95% CI
Age (≥60y)	0.913	0.996	0.318	2.491	0.415-14.957
Histological type	0.457	0.110	0.740	1.579	0.107-23.390
Grade	-1.369	3.599	0.058	0.254	0.062-1.046
Stage	-0.234	0.051	0.821	0.791	0.103-6.051
Tumor size	1.006	0.996	0.318	2.734	0.379-19.708
LN metastasis (+)	-0.061	0.004	0.948	0.941	0.154-5.735
Family history (+)	0.656	0.957	0.328	1.927	0.518-7.177
Menopause (+)	0.248	0.137	0.712	1.282	0.344-4.778
Her2 (+)	0.364	0.364	0.546	1.438	0.442-4.685
PR (+)	-0.17	0.000	0.984	0.983	0.185-5.220
ER (+)	0.024	0.001	0.978	1.024	0.188-5.578
Ki-67 (≥ 14%)	1.673	7.992	0.005	5.326	1.670-16.983
Jab1/COPS5	0.036	5.181	0.023	1.037	1.005-1.069

LN: lymph node.

### Association of Jab1/COPS5 expression with cancer patients

**Figure 4:**
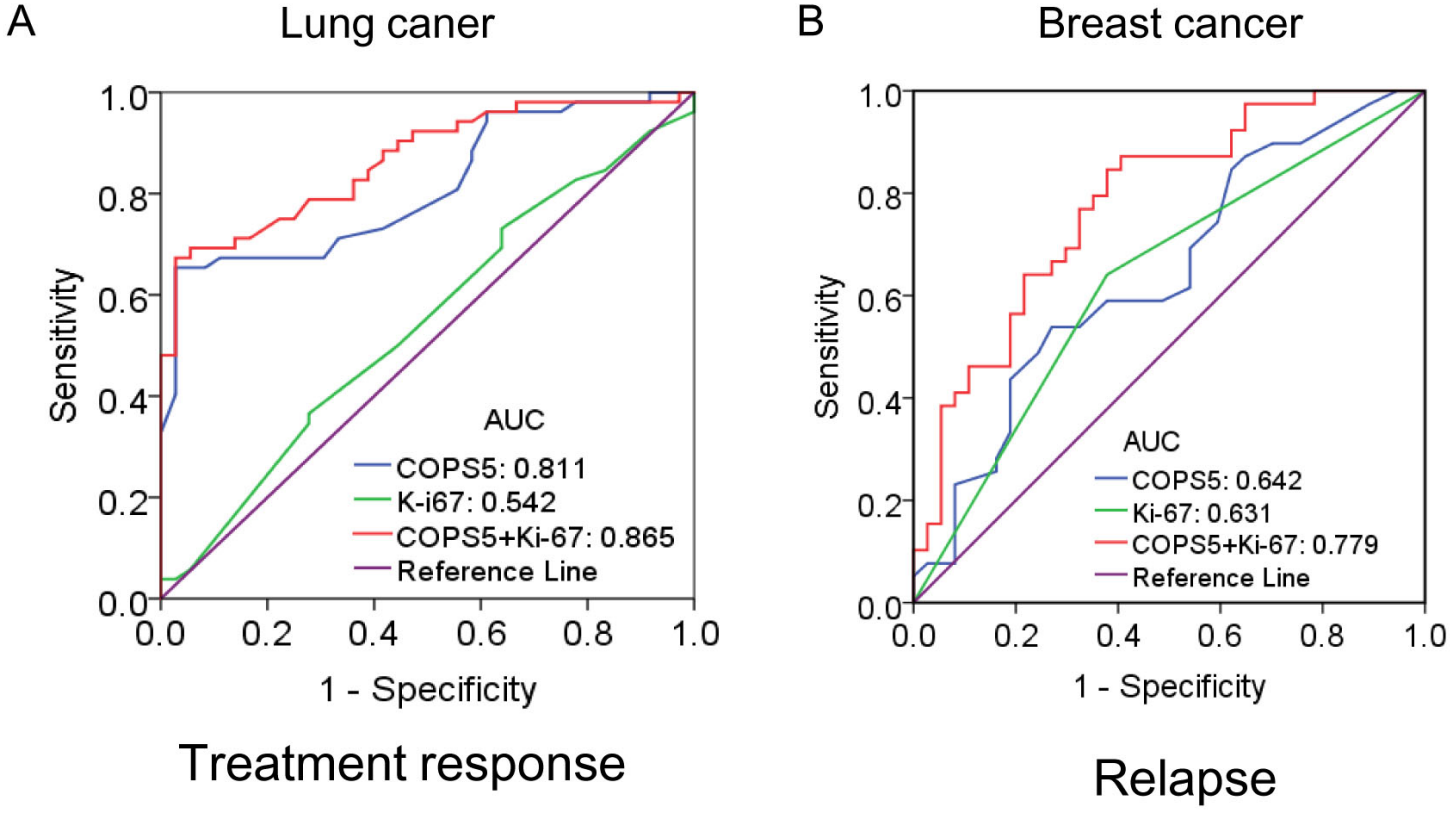
The diagnostic significance of Jab1/COPS5 was analyzed via establishing ROC curve in cancer The curve demonstrated that Jab1/COPS5 could discriminate between responder and non-responder of chemotherapy in lung cancer **(A)**, or non-relapse and relapse in breast cancer **(B)**.

To investigate the therapeutic value of COPS5 in lung cancer patients, ROC curve analysis was performed. As shown in Figure 4, the AUC value of ROC curve was 0.811, suggesting that COPS5 could distinguish the chemo-responsive patients from non-responsive patients. Combing COPS5 and Ki-67 would further enhance the therapeutic value with a higher AUC of 0.865 (Figure 4A).

The recrudescent value of COPS5 in breast cancer patients was also investigated. It was found the AUC value of ROC curve was 0.642, and the value (0.779) was further enhanced by combing COPS5 with Ki-67, indicating a potential role of COPS5 in distinguish primary tumors from relapse tumors (Figure 4B).

### Survival analysis

**Figure 5:**
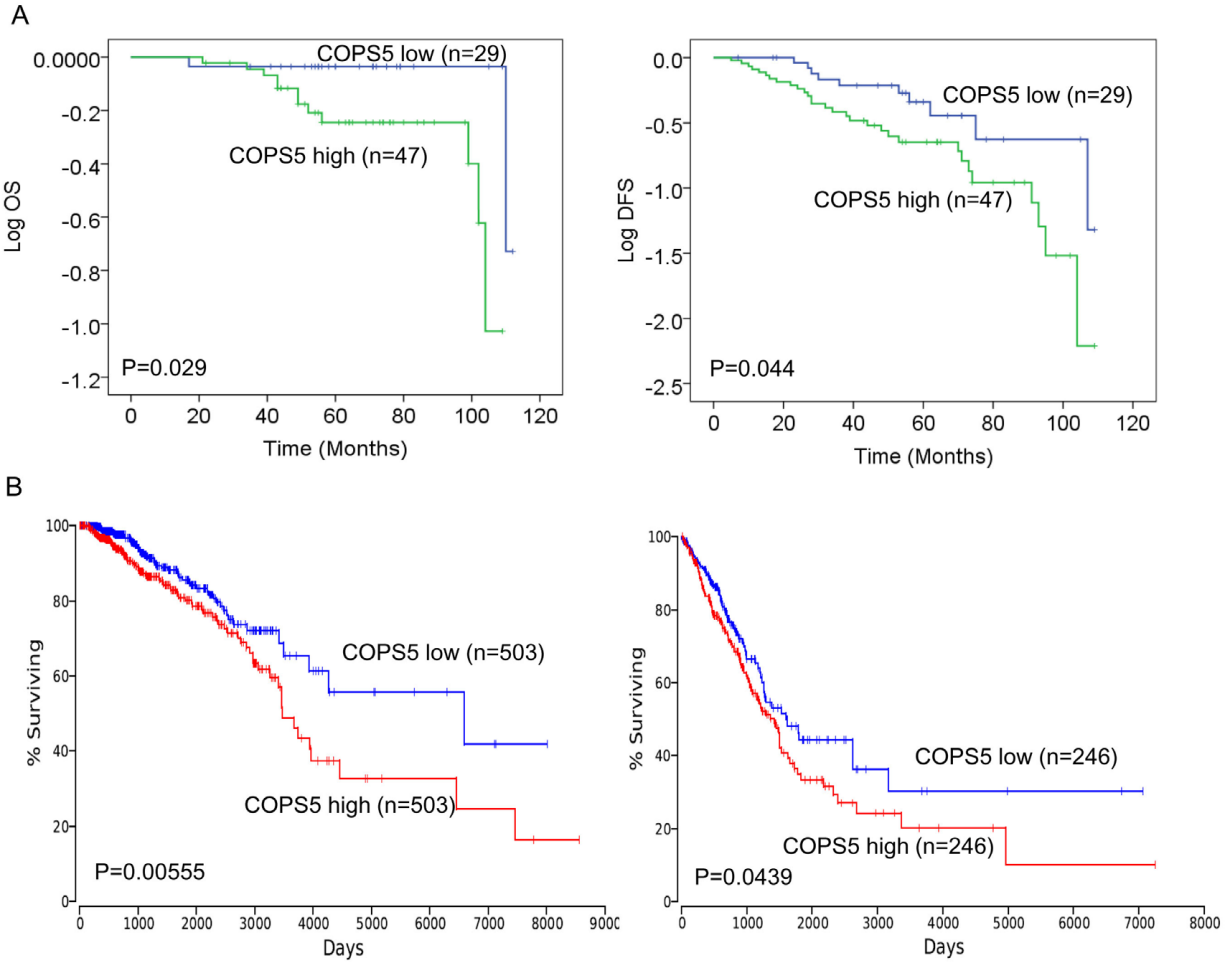
Jab1/COPS5 predicts survival in lung and breast cancer **(A)** Kaplan-Meier analyses of the association between Jab1/COPS5 protein expression and OS (left) and DFS (right) in breast cancer patients. **(B)** *In vivo* microarray data and Kaplan-Meier plots from OncoLnc database (http://www.oncolnc.org/) were used to assess correlations between Jab1/COPS5 gene expression and patient survival of lung cancer (left) and breast cancer (right).

The mean follow-up time of breast cancer patients was 66 months (17–120 (OS) and range 5-109 (DFS)). To evaluate the prognostic influence of Jab1/COPS5 expression on breast cancer patients, we performed Kaplan-Meier analyses to compare grouped patients. The survival curves suggested that patients with high levels of COPS5 had a significant association with worse DFS and OS in breast cancer (OS, p=0.029; DFS, p= 0.044; Figure 5A).

We also investigated the relationship between COPS5 and survival of cancer patients in OncoLnc database. The online data was consistent with the IHC data, suggesting high levels of COPS5 was associated with worse survival in either breast cancer (p=0.00555; Figure 5B) or lung cancer (p=0.0439; Figure 5B).

### Prognostic factors

To evaluate whether Jab1/COPS5 in breast cancer was independent predictor of DFS and OS, a multivariate analysis was performed with the following variables: age, histological type, tumor grade, tumor stage, tumor size, lymph node metastasis, Her2 status, ER status, PR status, menopause status, family history of cancer, Ki-67 and Jab1/COPS5. Histological type (p=0.017 for DSF; p=0.049 for OS) and COPS5 (p=0.001 for DSF; p=0.050 for OS) were significant prognostic factors for breast cancer (Table 5). Multivariate analysis identified COPS5 high expression as significant independent factor for poor DSF and OS in breast cancer.

**Table 5:** Multivariate survival analysis for DFS and OS in breast cancer

Parameters	DFS		OS	
	P	HR (95% CI)	P	HR (95% CI)
Age	0.725	0.79(0.21-2.93)	0.801	1.33(0.14-12.28)
Histological type	0.017	7.03(1.42-34.81)	0.049	12.57(1.01-155.87)
Tumor grade	0.478	0.71(0.28-1.82)	0.376	1.98(0.44-8.93)
Tumor stage	0.795	0.84(0.22-3.25)	0.489	3.30(0.11-96.9)
Tumor size	0.538	1.57(0.37-6.66)	0.965	0.93(0.03-25.06)
LN metastasis	0.987	0.99(0.33-3.02)	0.805	1.36(0.12-15.44)
Her2	0.683	1.18(0.53-2.66)	0.672	1.35(0.34-5.38)
ER	0.844	1.12(0.35-3.610	0.652	0.61(0.07-5.37)
PR	0.174	0.47(0.15-1.40)	0.846	0.82(0.11-6.28)
Menopause	0.711	1.21(0.45-3.26)	0.932	0.93(0.16-5.33)
Family history	0.420	1.40(0.62-3.20)	0.109	3.04(0.78-11.85)
Ki-67	0.059	1.99(0.98-4.10)	0.796	0.85(0.25-2.90)
Jab1/COPS5	0.001	1.03(1.01-1.05)	0.050	1.03(1.00-1.06)

DFS: disease-free survival; HR: hazard ratio; CI: confidence interval; LN: lymph node.

## DISCUSSION

The common development of chemotherapy resistance is a major determinant of poor outcome in lung cancer [17, 18]. Despite increased knowledge about drug resistance mechanisms [19–21], reliable biomarkers for prediction of therapy response or resistance in lung cancer patients have been lacking. Our study demonstrates that Jab1/COPS5 is upregulated in lung cancer tissue compared with non-cancerous tissue. High expression of Jab1/COPS5 protein in biopsy specimens of untreated primary tumors was associated with chemotherapy resistance, defined as non-responder after chemotherapy.

Increased levels of Jab1/COPS5 have previously been associated with shorter overall survival of lung cancer [22]. Our results suggest that Jab1/COPS5 is associated with lung cancer progression, for example, stage, metastasis and malignant hydrothorax. We also found that Jab1/COPS5 was an independent prognostic factor for treatment response and could be useful to identify platinum based chemotherapy resistant tumors prior to first-line systemic therapy. The prognostic value of Jab1/COPS5 was confirmed at the mRNA level by our analysis of independent publicly available microarray datasets, demonstrating a significant correlation between higher Jab1/COPS5 gene expression and reduced overall survival of lung cancer patients.

Derived from our findings that high protein levels of Jab1/COPS5 in primary untreated tumor specimens might help to identify lung cancer patients in whom chemotherapy resistance and the potentially improved efficacy of Jab1/COPS5-inhibition may balance toxicities and side effects of anti-cancer treatment.

In recent years, a number of studies revealed an association between elevated Jab1/COPS5 level and tumor progression/poor prognosis in several types of cancer, including breast cancer [10]. However, it lacks studies of Jab1/COPS5 in cancer patients with regard to chemotherapy response and cancer relapse. This study on breast cancer patients reveals that the changes in Jab1/COPS5 predict the DFS and OS; patients who had higher Jab1/COPS5 level had shorter DFS and OS than patients who had lower Jab1/COPS5 level. In addition, multivariate survival analyses showed that Jab1/COPS5 and histological type are independent prognostic factors for poor survival in patients. Increased Jab1/COPS5 levels in breast cancer can indicate cancer relapse, although the reason for why the elevated Jab1/COPS5 levels correlates with tumor relapse remains to be elucidated.

Accumulating evidence suggests critical roles of Jab1/COPS5 in breast cancer progression. A number of studies have demonstrated that Jab1 is overexpressed in breast cancer [23–25]. The involvement of Jab1 in breast cancer is linked to the ER, EGFR and HER2 pathways by mediating downstream signalling that contributes to the tumour progression [24, 25]. It was also proposed that ligand-induced ERα degradation was regulated by Jab1 in hormone-dependent breast cancer cells [26]. Recently, Lu R et. al., revealed that high levels of Jab1 are significantly enriched in the ERα+ breast cancer patients, implicating that the amplification and overexpression of COPS5 may contribute to the ERα-related signalling and tumour progression [10]. However, the clinical outcomes of the TCGA breast cancer patients with high expression levels of Jab1 appeared to be poorer regardless of tumour subtypes because Jab1 expression was similar in luminal A and B tumors [10]. In addition, Jab1 also interacts with both the progesterone receptor (PR) and the steroid receptor coactivator 1 (SRC-1) and that it stabilizes PR-SRC-1 complexes [27]. In line with these reports, we found Jab1 is overexpressed in human breast cancers, and in particular the ERα-, or PR- subset of breast cancer. However, the relationship between these biochemical indicators need to be further studied.

Our previous studies demonstrated that Stat3 [28] and non-coding RNAs [29] contribute to overexpression of Jab1/COPS5 in cancer, and highly expressed Jab1/COPS5 regulates DNA damage response, which confers chemotherapy and radiotherapy resistance to tumor cells [30]. Our previous animal studies also confirmed that suppression of Jab1/COPS5 signaling induces radio- and chemo-sensitivity in nasopharyngeal carcinoma [30]. The above evidence could support some results of this study that breast cancer patients with different Jab1/COPS5 levels have different relapse incidence.

In this study, we reproduced similar conclusion as previous studies that the non-responders to platinum based chemotherapy, tended to have increased Jab1/COPS5. Therefore, Jab1/COPS5 appears to indicate the response to platinum based chemotherapy. In addition, the Jab1/COPS5 level was also found to be an independent determining factor for DFS and OS. With detecting the Jab1/COPS5 expression, we could classify cancer patients into good and poor prognosis groups, select patients who have a high risk of postoperative relapse, and need strict follow-up and intense treatment.

In conclusion, we demonstrated that therapeutic resistant lung cancers and relapse breast cancers are characterized by distinct elevated of Jab1/COPS5. High intratumoral levels of Jab1/COPS5 might serve as novel predictive biomarkers to identify chemotherapy resistance in lung cancer or to identify relapse in breast cancer. Those cancer patients with high expression of Jab1/COPS5 might particularly benefit from additional target therapy.

## MATERIALS AND METHODS

### Patients and tissue samples

17 patients with pneumonia and 88 patients with lung cancer included in the study were from the First Affiliated Hospital of Zhengzhou University (Zhengzhou, Henan, PR China) from June 2016 to August 2016 (Table 1). 16 patients with breast hyperplasia and 76 patients with breast cancer treated at Anyang Tumor Hospital (Anyang, Henan, PR China) from August 2010 to July 2013 were included in this study. Patients were selected for this study on the basis of the availability of archived formalin-fixed paraffin-embedded tissue (FFPE) blocks for immunohistochemical analysis and administration of chemotherapy. However, in some patients surgical operation was performed and some patients were referred to other hospital, of whom FFPE could not be obtained. In total, 76 breast cancer patients including 37 non-recurrent tumors and 39 recurrent tumors were included as shown in Table 2. All of medical records were reviewed retrospectively. Ethical approval was obtained from the hospitals. Inquiries about the death were achieved by contacting the doctor-in-charge or their relatives. Written permission was obtained before surgery from all patients or their family.

### Treatment administration

Of the 88 lung cancer patients, 31 cases were small cell lung cancer (SCLC) and 57 cases were non-small cell lung cancer (NSCLC). In SCLC, 26 patients received platinum based double drug chemotherapy, 5 received radiotherapy plus chemotherapy. In NSCLC, 2 received gefitinib targeted therapy, 16 underwent surgery plus chemotherapy, and 39 received platinum based double drug chemotherapy. Platinum based chemotherapy was repeated twice every 3 weeks for 4-6 cycles. Before the third cycle, we evaluated treatment response use Response Evaluation Criteria in Solid Tumors (RECIST) version 1.1 for the first time and divided patients into four groups (complete response (CR), partial response (PR), stable disease (SD) and progressive disease (PD)) before surgery. We further divided the patients into responder (CR and PR) and non-responder (SD and PD).

Of the 76 breast cancer patients, 37 cases were primary tumors and were given surgical treatment and chemotherapy, of which 9 cases received combined radiotherapy and 1 case received endocrine treatment. 39 relapse patients were initially treated with surgery, of which 32 cases received chemotherapy in the initial and relapsed period, 6 cases were treated with adjuvant chemotherapy only after the initial treatment and 1 patient received preoperative chemotherapy and chemotherapy in the initial and relapsed period. The chemotherapy included anthracycline and taxane drugs.

### Hematoxylin and eosin (HE) staining and immunohistochemical analysis

The tissues were routinely stained with hematoxylin and eosin (HE). The histological characteristics were reviewed by two pathologists. The Jab1/COPS5 levels in the FFPE tissue sections were detected by immunohistochemical analysis [28]. Briefly, the specimens were sectioned and mounted on Superfrost/Plus slides (Fisher Scientific, Pittsburgh, PA), and then deparaffinized in two xylene. The slides were boiled in 0.01 mol/L sodium citrate (pH6.0) with a pressure cooker at maximum heat for 3 minutes for antigen retrieval. The slides were incubated with the primary antibody Jab1/COPS5 (Santa Cruz, sc-13157) at 1:100 dilution overnight. The sections were incubated with secondary antibody and 3,3-diaminobenzidine tetrahydro-chloride (DAB). The slides were then counterstained with hematoxylin. Jab1/COPS5 levels were examined by counting at least 200 tumor cells in representative high-power fields. All experiments were performed in accordance with approved guidelines and regulations of Anyang Tumor Hospital.

### Analysis of clinical mRNA microarrays for the detection of correlations between Jab1/COPS5 and patients survival

Microarray analysis and visualization platform R2 (http://r2.amc.nl) was used to determine whether the expression levels of Jab1/COPS5 were correlated with the treatment response of lung cancer, or with the relapse status of breast cancer. We specifically used the tumor lung adenocarcinoma dataset (Tumor Lung Adenocarcinoma - TCGA - 515 - rsem - tcgars) and tumor breast dataset (Tumor Breast – Wang – 286 MAS5.0 - u133A) and selected the analysis with “View a gene”.

OncoLnc database (http://www.oncolnc.org/) was used to determine whether the expression of Jab1/COPS5 is correlated with the overall survival of lung cancer and breast cancer. We specifically sumitted COPS5 gene and plot kaplan with “LUAD (Lung adenocarcinoma)” and “BRCA (Breast invasive carcinoma”.

### Statistical analysis

Relapse of breast cancer was defined as the regional or distant relapse in any site. Disease-free survival (DFS) was defined as the time from initial diagnosis to relapse, metastasis or death. Overall survival (OS) was calculated as the period from initial diagnosis to death attributable to any cause.

Chi-Square test was used to compare the expression of Jab1/COPS5 with various clinicopathologic variables. Statistical analysis for the results was analyzed using Mann-Whitney test when only two groups. Logistic regression was used in multivariate analyses to identify risk factors impacting treatment response and relapse. The receiver operating characteristic (ROC) curves was generated to evaluate the therapeutic and recrudescent performance of Jab1/COPS5 in cancer. Kaplan-Meier method was used to plot the survival curves and differences between the survival curves were detected by log-rank test. P <0.05 was considered significant. The statistical analyses were performed using SPSS version 22.0 software (SPSS, Chicago, IL).

## SUPPLEMENTARY MATERIALS TABLE



## Abbreviations

EGFR: epidermal growth factor receptor
HR: hormone receptor
HER2: human epidermal growth factor receptor 2
ER: estrogen
PR: progesterone receptor
DDR: DNA damage response
NSCLC: non-small-cell lung cancer
SCLC: small cell lung cancer
TNBC: triple negative breast cancers
HE: hematoxylin and eosin
DFS: disease-free survival
OS: overall survival

## References

[1] Maitland ML, Schilsky RL (2011). Clinical trials in the era of personalized oncology. CA Cancer J Clin.

[2] Liu Y, Zhang Y, Zhang L, Liu B, Wang Y, Zhou X, Li Y, Zhao Q, Gong Y, Zhou L, Zhu J, Ding Z, Wang J (2017). Efficacy of epidermal growth factor receptor-tyrosine kinase inhibitors for lung squamous carcinomas harboring EGFR mutation: a multicenter study and pooled analysis of published reports. Oncotarget.

[3] Cocciolone V, Cannita K, Calandrella ML, Ricevuto E, Baldi PL, Sidoni T, Irelli A, Paradisi S, Pizzorno L, Resta V, Bafile A, Alesse E, Tessitore A (2017). Prognostic significance of clinicopathological factors in early breast cancer: 20 years of follow-up in a single-center analysis. Oncotarget.

[4] Kourea HP, Zolota V, Scopa CD (2014). Targeted pathways in breast cancer: molecular and protein markers guiding therapeutic decisions. Curr Mol Pharmacol.

[5] Claret FX, Hibi M, Dhut S, Toda T, Karin M (1996). A new group of conserved coactivators that increase the specificity of AP-1 transcription factors. Nature.

[6] Pan Y, Yang H, Claret FX (2014). Emerging roles of Jab1/CSN5 in DNA damage response, DNA repair, and cancer. Cancer Biol Ther.

[7] Pan Y, Zhang Q, Tian L, Wang X, Fan X, Zhang H, Claret FX, Yang H (2012). Jab1/CSN5 negatively regulates p27 and plays a role in the pathogenesis of nasopharyngeal carcinoma. Cancer Res.

[8] Guo H, Jing L, Cheng Y, Atsaves V, Lv Y, Wu T, Su R, Zhang Y, Zhang R, Liu W, Rassidakis GZ, Wei Y, Nan K (2016). Down-regulation of the cyclin-dependent kinase inhibitor p57 is mediated by Jab1/Csn5 in hepatocarcinogenesis. Hepatology.

[9] Wan M, Cao X, Wu Y, Bai S, Wu L, Shi X, Wang N, Cao X (2002). Jab1 antagonizes TGF-beta signaling by inducing Smad4 degradation. EMBO Rep.

[10] Lu R, Hu X, Zhou J, Sun J, Zhu AZ, Xu X, Zheng H, Gao X, Wang X, Jin H, Zhu P, Guo L (2016). COPS5 amplification and overexpression confers tamoxifen-resistance in ERalpha-positive breast cancer by degradation of NCoR. Nat Commun.

[11] Zhou F, Pan Y, Wei Y, Zhang R, Bai G, Shen Q, Meng S, Le XF, Andreeff M, Claret FX (2017). Jab1/Csn5-thioredoxin signaling in relapsed acute monocytic leukemia under oxidative stress. Clin Cancer Res.

[12] Pan Y, Claret FX (2012). Targeting Jab1/CSN5 in nasopharyngeal carcinoma. Cancer Lett.

[13] de Groot P, Munden RF (2012). Lung cancer epidemiology, risk factors, and prevention. Radiol Clin North Am.

[14] Gadgeel SM, Ramalingam SS, Kalemkerian GP (2012). Treatment of lung cancer. Radiol Clin North Am.

[15] Chen L, Linden HM, Anderson BO, Li CI (2014). Trends in 5-year survival rates among breast cancer patients by hormone receptor status and stage. Breast Cancer Res Treat.

[16] Esteva FJ, Sahin AA, Rassidakis GZ, Yuan LX, Smith TL, Yang Y, Gilcrease MZ, Cristofanilli M, Nahta R, Pusztai L, Claret FX (2003). Jun activation domain binding protein 1 expression is associated with low p27(Kip1)levels in node-negative breast cancer. Clin Cancer Res.

[17] Visconti R, Morra F, Guggino G, Celetti A (2017). The between now and then of lung cancer chemotherapy and immunotherapy. Int J Mol Sci.

[18] Armas-Lopez L, Pina-Sanchez P, Arrieta O, Guzman de Alba E, Ortiz-Quintero B, Santillan-Doherty P, Christiani DC, Zuniga J, Avila-Moreno F (2017). Epigenomic study identifies a novel mesenchyme homeobox2-GLI1 transcription axis involved in cancer drug resistance, overall survival and therapy prognosis in lung cancer patients. Oncotarget.

[19] Tripathi SC, Fahrmann JF, Celiktas M, Aguilar M, Marini KD, Jolly MK, Katayama H, Wang H, Murage EN, Dennison JB, Watkins DN, Levine H, Ostrin EJ (2017). MCAM mediates chemoresistance in small cell lung cancer via the PI3K/AKT/SOX2 signaling pathway. Cancer Res.

[20] Cheng W, Liang C, Xu L, Liu G, Gao N, Tao W, Luo L, Zuo Y, Wang X, Zhang X, Zeng X, Mei L (2017). TPGS-functionalized polydopamine-modified mesoporous silica as drug nanocarriers for enhanced lung cancer chemotherapy against multidrug resistance. Small.

[21] Xiao L, Lan X, Shi X, Zhao K, Wang D, Wang X, Li F, Huang H, Liu J (2017). Cytoplasmic RAP1 mediates cisplatin resistance of non-small cell lung cancer. Cell Death Dis.

[22] Goto A, Niki T, Moriyama S, Funata N, Moriyama H, Nishimura Y, Tsuchida R, Kato JY, Fukayama M (2004). Immunohistochemical study of Skp2 and Jab1, two key molecules in the degradation of P27, in lung adenocarcinoma. Pathol Int.

[23] Kouvaraki MA, Rassidakis GZ, Tian L, Kumar R, Kittas C, Claret FX (2003). Jun activation domain-binding protein 1 expression in breast cancer inversely correlates with the cell cycle inhibitor p27(Kip1). Cancer Res.

[24] Wang J, Barnes RO, West NR, Olson M, Chu JE, Watson PH (2008). Jab1 is a target of EGFR signaling in ERalpha-negative breast cancer. Breast Cancer Res.

[25] Hsu MC, Chai CY, Hou MF, Chang HC, Chen WT, Hung WC (2008). Jab1 is overexpressed in human breast cancer and is a downstream target for HER-2/neu. Mod Pathol.

[26] Callige M, Kieffer I, Richard-Foy H (2005). CSN5/Jab1 is involved in ligand-dependent degradation of estrogen receptor {alpha} by the proteasome. Mol Cell Biol.

[27] Chauchereau A, Georgiakaki M, Perrin-Wolff M, Milgrom E, Loosfelt H (2000). JAB1 interacts with both the progesterone receptor and SRC-1. J Biol Chem.

[28] Pan Y, Wang S, Su B, Zhou F, Zhang R, Xu T, Zhang R, Leventaki V, Drakos E, Liu W, Claret FX (2017). Stat3 contributes to cancer progression by regulating Jab1/Csn5 expression. Oncogene.

[29] Wang S, Pan Y, Zhang R, Xu T, Wu W, Zhang R, Wang C, Huang H, Calin CA, Yang H, Claret FX (2016). Hsa-miR-24-3p increases nasopharyngeal carcinoma radiosensitivity by targeting both the 3’UTR and 5’UTR of Jab1/CSN5. Oncogene.

[30] Pan Y, Zhang Q, Atsaves V, Yang H, Claret FX (2013). Suppression of Jab1/CSN5 induces radio- and chemo-sensitivity in nasopharyngeal carcinoma through changes to the DNA damage and repair pathways. Oncogene.

