# Supplementary material for: Increased Jab1/COPS5 is associated with therapeutic response and adverse outcome in lung cancer and breast cancer patients
Source: Oncotarget. 2017 Oct 27;8(57):97504–15. doi: 10.18632/oncotarget.22146 (PMC5722579; doi:10.18632/oncotarget.22146)
Supplement: Supplementary file 1 [file oncotarget-08-97504-s001.pdf]

Increased Jab1/COPS5 is associated with therapeutic response and adverse outcome in lung cancer and breast cancer patients

SUPPLEMENTARY MATERIALS

Supplementary Table 1: Characteristics of noncancerous and cancerous patients

Variable	Lung cancer	Pneumonia	P	Breast cancer	Hyperplasia	P
Age						
<60	39(44.3%)	8(47.1%)	0.99	64(84.2%)	12(75%)	0.47
≥60	49(55.7%)	9(52.9%)		12(15.8%)	4(25%)	
Sex						
M	67(76.1%)	11(64.7%)	0.37			
F	21(23.9%)	6 (35.3%)				
Smoking						
No	37 (42.0%)	11 (64.7%)	0.11			
Yes	51(58.0%)	6 (35.3%)				
